# The Ciji-Hua’ai-Baosheng II Formula Attenuates Chemotherapy-Induced Anorexia in Mice With H_22_ Hepatocellular Carcinoma

**DOI:** 10.3389/fphar.2021.715824

**Published:** 2021-08-19

**Authors:** Shengyan Xi, Xiangyang Zhai, Yanan Wang, Yuewen Gong, Biqian Fu, Chunling Gao, Xuehui Guo, Yunhong Li, Zheng Wang, Shuqiong Huang, Dawei Lu, Yufang Zhao, Linchao Qian, Yanhui Wang

**Affiliations:** ^1^ Department of Traditional Chinese Medicine, School of Medicine, Xiamen University, Xiamen, China; ^2^ Department of Traditional Chinese Medicine, Xiang’an Hospital of Xiamen University, Xiamen, China; ^3^ College of Pharmacy, Rady Faculty of Health Sciences, University of Manitoba, Winnipeg, MB, Canada; ^4^ The First School of Clinical Medicine, Guangzhou University of Chinese Medicine, Guangzhou, China; ^5^ Department of Radiotherapy, Chenggong Hospital of Xiamen University, Xiamen, China

**Keywords:** Ciji-Hua’ai-Baosheng II formula, traditional Chinese medicine, hepatocellular carcinoma, chemotherapy, anorexia

## Abstract

**Background:** Ciji-Hua’ai-Baosheng II Formula (CHB-II-F) is a traditional Chinese medicine formula, which specifically targets different aspects of chemotherapy-induced adverse effects in patients with cancer. In our clinical application, CHB-II-F significantly alleviated chemotherapy-induced anorexia (loss of appetite) and improved the quality of life for patients with tumor during and after chemotherapy. However, the mechanism of CHB-II-F in alleviation of chemotherapy-induced anorexia remains to be further investigated.

**Aim of Study:** To explore the therapeutic effect and mechanism of CHB-II-F on chemotherapy-induced anorexia in the mice model of H_22_ hepatoma.

**Materials and Methods:** A total of 72 Kunming mice of SPF grade were inoculated subcutaneously with H_22_ hepatoma cells into the right anterior armpit of the mice. After 1 week of seeding, mice were injected intraperitoneally with a high dose of 5-fluorouracil (200 mg/kg 5-FU) to establish the model of chemotherapy. The mice were randomly divided into six groups: untreated group, 5-FU group, 5-FU plus Yangzheng Xiaoji capsule (YZXJC) group, and three groups of 5-FU plus different concentrations of CHB-II-F. All the mice in each group were treated for 14 days. The body weight, food intake, tumor volume, and tumor weight of mice were measured, and pathological examinations of tumor tissue, stomach, and duodenum were carried out. Expressions of serum Leptin, Neuropeptide Y (NPY), epidermal cell growth factor (EGF), Motilin (MTL), Orexin A (OXA), Gastrin (GAS), Ghrelin, Prostaglandin E_2_ (PGE_2_), and jejunum superoxide dismutase (SOD) activity and malondialdehyde (MDA) content were examined. The protein and mRNA levels of proopiomelanocortin (POMC), Orexin receptor 1 (OX1R), neuropeptide Y (NPY), cocaine and amphetamine regulated transcript peptide (CART), Agouti gene-related protein (AgRP), Leptin receptor (Ob-R), and Ghrelin receptor (GHSR) were examined in hypothalamus, and the protein levels of substance P (SP) and 5-hydroxytryptamine (5-HT) in duodenum were measured.

**Results:** The combination of CHB-II-F and 5-FU could enhance the inhibitory effect of 5-FU on tumor. The tumor inhibition rates of 5-FU group, YZXJC group, CHB-II-F(H) group, CHB-II-F(M) group, and CHB-II-F(L) group were 58.88, 28.08, 54.96, 37.69, and 28.61%, respectively. Compared with untreated group and 5-FU group, CHB-II-F significantly increased the body weight and food intake of tumor-bearing mice; increased the content of NPY, Orexin A, Ghrelin, GAS, MTL, EGF, and PGE_2_ in serum and the activity of SOD in jejunum; and decreased the content of Leptin in serum and the content of MDA in jejunum. Compared with untreated group and 5-FU group, CHB-II-F also enhanced the expression of OX1R, GHSR, NPY, and AgRP protein and gene and decreased the expression of Ob-R, POMC, and CART protein and gene in hypothalamus of mice, and the gene expression was consistent with the protein expression. In addition, CHB-II-F decreased the expression of 5-HT and SP protein in duodenum.

**Conclusion:** In the murine model of H22 hepatocellular carcinoma (HCC) receiving chemotherapy, CHB-II-F enhances the inhibitory effect of 5-FU on tumor, significantly improves the pathological injury of gastrointestinal tract caused by chemotherapy, and regulates the secretion of gastrointestinal hormones. It may alleviate chemotherapy-induced anorexia by affecting appetite regulatory factors in the feeding area of hypothalamus central nervous system and peripheral appetite regulatory factors.

## Introduction

Cancer is the second leading cause of death globally only after cardiovascular disease (Fitzmaurice et al., 2017). The incidence of liver cancer ranks the sixth in the global incidence of cancer, ranking the fourth among cancer deaths, and the number of deaths from liver cancer in 2015 was about 810,000 (GBD 2015 Mortality and Causes of Death, 2016). Surgical resection and liver transplantation are usually the effective treatment methods for early stage of liver cancer, but most of the patients are in middle and late stages when diagnosed and are no longer suitable for surgical resection, and palliative chemotherapy is the commonly used treatment at these stages (Zhang et al., 2018). In most cases, targeting the characteristics of unlimited proliferation and abnormal differentiation of malignant tumor cells, chemotherapy can usually kill cancer cells or inhibit the growth and differentiation of cancer cells (Ku, 2017). However, the cytotoxic effect of chemotherapeutic drugs is not selective. While it causes damage to tumor cells, normal tissue cells also suffer from varying degrees of injury, especially gastrointestinal mucosal epithelial cells due to their active growth. The most common symptoms are nausea, vomiting, anorexia (loss of appetite), diarrhea, and oral and intestinal mucositis (Shiomi et al., 2018). These gastrointestinal complications caused by chemotherapy aggravate the injury of patients after chemotherapy, resulting in a serious decline in the quality of life and a decrease in compliance of patients, thus hindering further chemotherapy for patients in clinic (Akin et al., 2010; Iwasa et al., 2011; Rojas and Slusher, 2012; Oteki et al., 2016).

Chemotherapy-induced nausea and vomiting (CINV) is one of the most common side effects of gastrointestinal toxicity caused by chemotherapy. Pathogenesis of CINV may include both peripheral and central nervous system (CNS). According to the occurrence of CINV, CINV is often divided into acute CINV, refractory CINV, expectant CINV, breakthrough CINV, and delayed CINV (Cohen et al., 2007). In acute CINV, free radicals generated by chemotherapy stimulate enterochromaffin cells in the gastrointestinal tract, causing the release of serotonin. Serotonin binds to intestinal vagal afferent nerves *via* 5-hydroxytryptamine 3 (5-HT3) receptors, which trigger the vomiting reflex via the nucleus of the solitary tract (NTS) and chemoreceptor trigger zone (CTZ) in the CNS. Substance P is considered to be the principal neurotransmitter involved in delayed CINV. Chemotherapy triggers the release of substance P from neurons in the central and peripheral nervous systems, which then binds to neurokinin-1 (NK-1) receptors mainly in the NTS to induce vomiting (Janelsins et al., 2013; Rapoport, 2017). Although modern medicine has made great progress in the prevention and treatment of CINV and even with the number of cases has been greatly reduced with the widespread use of NK-1 receptor antagonists and 5-HT3 receptor antagonists in clinical practice, there are still some problems in the clinical treatment of CINV (Salihah et al., 2016). At this time, chemotherapy-induced loss of appetite becomes a prominent symptom of side effects of alimentary tract caused by chemotherapy. Improper treatment will lead to malnutrition, weakened resistance, and even secondary infection that causes rapid deterioration of the patients’ condition. However, no effective drugs have been developed to treat the chemotherapy-induced anorexia (Oteki et al., 2016), although corticosteroids such as dexamethasone and megestrol can promote appetite to a certain extent, but it is difficult to be widely used because of their many side effects.

In recent years, traditional Chinese medicine formulation has been widely used in various aspects of tumor treatment and especially in the treatment of chemotherapy-induced loss of appetite with good efficacy and few side effects, so it has attracted many attentions (Hu et al., 2015; Xue et al., 2011). At present, there are many clinical reports on the treatment of chemotherapy-induced anorexia by traditional Chinese medicine formulation with different clinical effects, but the overall therapeutic efficacy still needs to be further improved. The reason of anorexia caused by chemotherapy in malignant tumor is very complicated, and the main reason may be the direct stimulation of gastrointestinal mucosa by chemotherapeutic drugs, which may lead to inflammation in the gastrointestinal tract. Meanwhile, chemotherapeutic drugs inhibit and damage the proliferating gastrointestinal mucosa cells, which results in abnormal gastrointestinal secretion and decreases digestive enzyme activity and gastrointestinal functions of digestion and absorption.

5-FU is one of the most commonly used chemotherapeutic drugs in clinic at present. It is a classic drug for the treatment of malignant tumors of the digestive tract (Longley et al., 2003). It has a good efficacy for liver cancer, cholangiocarcinoma, gastric cancer, colon cancer, rectal cancer, and other digestive tract tumors. However, side effects including gastrointestinal toxicity, hepatorenal toxicity, and digestive tract mucositis restrict its clinical application (Thongprasert, 2005). In China, doctors in traditional Chinese medicine usually combine Chinese medicine with 5-FU to treat patients with tumor. The traditional Chinese medicine can not only enhance the antitumor effect of 5-FU, but also alleviate many toxic and side effects caused by 5-FU and, therefore, significantly improve the patient’s tolerance to chemotherapy. Yangzheng Xiaoji capsule (YZXJC) is an antitumor Chinese herbal formulation commonly used in clinic. It can enhance the antitumor effect of chemotherapeutic drugs and relieve the damage of chemotherapeutic drugs to the gastrointestinal tract, immune function, and hematopoiesis of the patients. Therefore, in this study, 5-FU was used as chemotherapy drug and Yangzheng Xiaoji capsule was selected as control formulation of traditional Chinese medicine.

Ciji-Hua’ai-Baosheng Decoction (CHBD) is an empirical prescription formulated by Professor Wang Yanhui of Xiamen University School of Medicine and Xiang’an Hospital for long-term care of living and improvement of quality of life after chemotherapy. The clinical application of CHBD proved to be very effective. Early investigation showed that CHBD combined with Cyclophosphamide (CTX) could enhance the inhibitory effect of CTX on the proliferation and growth of tumor cells; prolong the survival time of mice after tumor chemotherapy; increase the numbers of leukocytes, platelets, and erythrocytes in mice after tumor chemotherapy; and alleviate the suppression of bone marrow hematopoietic stem cell differentiation induced by tumor chemotherapy (Xi et al., 2014). In addition, CHBD can improve the immune function of mice after tumor chemotherapy and alleviate the colon inflammatory reaction induced by CTX chemotherapy (Xi et al., 2018).

In order to further develop a traditional Chinese medicine formulation for adjusting the internal environment of cancer and human body, Professor Wang Yanhui refined 19 Chinese herbs into eight herbs while keeping the basic treatment principles of CHBD unchanged to form CHB-II-F. Previous animal experiments showed that CHB-II-F had little side effect and could increase the weight of mice after tumor chemotherapy. In combination with 5-FU, the inhibition of tumor cells may be enhanced by promoting apoptosis of tumor cells and regulating the expression of tumor apoptosis factors (Fu et al., 2018; Fu et al., 2019). The purpose of this study was to explore the therapeutic effect and mechanism of CHB-II-F on loss of appetite after chemotherapy in H_22_ transplanted tumor mice and to provide more experimental evidence for its clinical application.

## Materials and Methods

### Experimental Animals and Tumor Cells

A total of 72 specific pathogen-free (SPF) Kunming mice of 6-weeks-old with 50% female and 50% male and weighing 20 ± 2 g were purchased from SLAC Laboratory Animal Co. Ltd. in Shanghai, China [license no. SCXK (Hu) 2017-0005]. All the mice were randomly divided into six groups with six mice in each group and the same numbers of male and female. The mice were adapted in the SPF class animal room of the Experimental Animal Center of Xiamen University [license no. SYXK (Min) 2018-0009] with room temperature of 22 ± 2°C, air humidity of 55 ± 20%, light and dark cycle of 12 h, and ad libitum feeding for 1 week before the experiment. The H_22_ hepatoma cell suspension was supplied from the ascites of H_22_ hepatoma bearing mice by Experimental Animal Center of Xiamen University. After 1-week subculture in ascites, the experiments were carried out in accordance with the regulation of the Laboratory Animal Administration and Ethics Committee of Xiamen University (no. XMULAC20170018).

### Experimental Drugs

CHB-II-F is composed of Radix Salviae Miltiorrhizae, Radix Codonopsis, Fructus Hordei Germinatus, Poria, Concha Ostreae, Bulbus Fritillariae Ussuriensis, Pericarpium Citri Reticulatae, and Semen Ziziphi Spinosae (Table 1). All of the above medicinals were purchased from the Yanlaifu Pharmaceutical Co., Ltd. (Xiamen, China). The maximum tolerated dose (MTD) of CHB-II-F on mice was 94.31 g/kg [351 g (dried medicinal herb)/kg], and mouse median lethal dosage (LD 50) could not be measured due to the low toxic effect of CHB-II-F (Fu et al., 2019). Its chemical fingerprint of CHB-II-F was analyzed by ultra-high performance liquid chromatography (UHPLC). Yangzheng Xiaoji capsule with a product lot number of A1711003 containing 0.39 g in each capsule was produced by Shijiazhuang Yiling Pharmaceutical Co., Ltd. (Shijiazhuang, China). 5-Fluorouracil (5-FU) injection of 250 mg (10 ml) per ampoule with a product lot number of 1707091 was produced by Tianjin Jinyao Pharmaceutical Co., Ltd. (Tianjin, China). 0.9% of sodium chloride (product lot no. 1803152) containing 100 ml in each ampoule was produced by Fujian Tianquan Pharmaceutical Co., Ltd. (Fujian, China).

**TABLE 1:** Information of components in CHB-II-F.

Chinese name	Botanical name	Common name	Weight (g)	Voucher numbers	Part used
Dan Shen	*Salvia miltiorrhiza* Bge	Radix Salviae Miltiorrhizae	50	170,815	Root and rhizome
Dang Shen	*Codonopsis pilosula* (Franch.) Nannf. *Codonopsis pilosula* Nannf. var. *modesta* (Nannf.) L.T. Shen or *Codonopsis tangshen* Oliv	Radix Codonopsis Pilosulae	10	170,405	Root and rhizome
Fu Ling	*Poria cocos* (Schw.) Wolf	Sclerotium Poriae Cocos	30	170,207	Sclerotium
Chen Pi	*Citrus reticulata* Blanco	Pericarpium Citri Reticulatae	10	170,809	Matured pericarp
Mai Ya	*Hordeum vulgare* L.	Fructus Hordei Germinatus	20	170,704	Germinated matured fruit
Suan Zao Ren	*Ziziphus jujuba* Mill. var. *spinosa* (Bunge) Hu ex H. F. Chou	Semen Ziziphi Spinosae	25	170,711	Matured seed
Mu Li	*Ostrea gigas* Thunberg, *Ostrea talienwhanensis* Crosse or *Ostrea rivularis* Gould	Concha Ostreae	20	170,701	Shell
Ping Bei Mu	*Fritillaria ussuriensis* maxim	Bulbus Fritillariae Ussuriensis	30	170,516	Squamous bulb

### Main Reagents and Kits

In this study, the following reagents were purchased: MLGR-E21637 mouse SOD ELISA kit (product lot no. ml001998), MLGR-E20206 mouse MDA ELISA kit (product lot no. ml931407), MLGR-E20561 mouse PGE_2_ ELISA kit (product lot no. ml037542), MLGR-E20203 mouse EGF ELISA kit (product lot no. ml297701), MLGR-E20682 mouse MTL ELISA kit (product lot no. ml201829), MLGR-E20684 mouse GAS ELISA kit (product lot no. ml037432), MLGR-E27958 mouse Ghrelin ELISA kit (product lot no. ml037434), and MLGR-E20624 mouse Leptin ELISA kit (product lot no. ml002287) were all purchased from the Enzyme-Linked Biotechnology Co., Ltd. (Shanghai, China). NPY antibody (product lot no. ab180809), AgRP antibody (product lot no. ab194645), POMC antibody (product lot no. ab94446), and CART antibody (product lot no. ab192364) were all purchased from the Abcam Company (Cambridge, United Kingdom). OX1R antibody (product lot no. 18370-1-AP) was purchased from the Proteintech Group, Inc. (Wuhan, China). Ob-R antibody (product lot no. sc-8391) was purchased from the Santa Cruz Biotechnology, Inc. (Dallas, United States). Rabbit anti-GHSR antibody (product lot no. D160429) was purchased from the Sangon Biotech Co., Ltd. (Shanghai, China). Rabbit anti-substance P antibody (product lot no. bs-0065R), rabbit anti-5-HT antibody (product lot no. bs-1126R), rabbit anti-Orexin receptor 1 antibody (product lot no. bs-18029R), rabbit anti-Leptin receptor antibody (product lot no. bs-0961R), and rabbit anti-Ghrelin receptor antibody (product lot no. bs-11529R) were purchased from the Bioss Biotechnology Co., Ltd. (Beijing, China). RNA extraction kit (product lot no. RN0401) was purchased from the Edley Biotechnology Co., Ltd. (Beijing, China).

### Main Instruments

The following instruments were employed in the experiment: FreeZone-18 freeze dryer (Labconco Co., Kansas City, United States), ASP200S automatic tissue dehydrator (LEICA Co., Solms, Germany), BX53 intelligent biomicroscope (Olympus optical Co. Ltd., Tokyo, Japan), Leica RM2245 paraffin slicer (LEICA Co., Solms, Germany), GEL Doc XR Gel Imager (Bio-Rad Inc., California, United States), 85–2 magnetic mixer (Changzhou Guohua Instruments Co., Ltd., Changzhou, China), LDZX-50KBS high pressure steam sterilization pot (Shanghai Shenan Medical Device Factory, Shanghai, China), R-134A high-speed freeze centrifuge (Eppendorf Inc., Hamburg, Germany), EG1150H embedding machine (LEICA Co., Solms, Germany), Victor3V multifunction enzyme marker (PerkinElmer, Inc., San Francisco, California, United States), StepOnePlus real-time fluorescence quantitative PCR (Applied Biosystems Inc., California, United States), IMS-100 Ice Machine (Changshu Xueke Electric Appliances Co., Ltd., Changshu, China), and PowerPac Basic Gel Electrophoresis instrument (Bio-Rad Inc., California, United States).

### Medicinal Preparation

A total of 195 g of CHB-II-F crude herb was mixed and immersed in a container for 20 min. After that 1950 ml of water was added and boiled for 30 min, and the liquid was filtered with eight layers of gauze. After adding 1500 ml water to the residue and boiling 30 min again, the liquid was filtered again with eight layers of gauze. Two filtered liquids were combined and condensed to 60 ml (3.25 g/ml) at 58°C on rotating evaporator (Shanghai Yarong Biochemistry Instrument Factory, Shanghai, China) and then lyophilized with freezer-dryer (Beijing Songyuan Huaxing Technology Development Co., Ltd., Beijing, China). The weight of the final freeze-dried powder of CHB-II-F (195 g) in each preparation was 52.4 g. The extraction yield was 26.87%. The freeze-dried powder was stored in the refrigerator at 4°C until experiment. In the experiment, the freeze-dried powder was diluted into 3.25 g/ml (high dose of CHB-II-F), 1.625 g/ml (medium dose of CHB-II-F), and 0.8125 g/ml (low dose of CHB-II-F), respectively, with distilled water and stored in refrigerator at 4°C. 5-FU was diluted with 0.9% sodium chloride solution, and the concentration was adjusted to 10 mg/ml and 1 mg/ml. The concentration of YZXJC was diluted to 0.039 g/L with distilled water.

### UHPLC-MS

UHPLC was combined with high-resolution electrospray ionization mass (HR-ESI-MS) detector to analyze the active constituents in CHB decoction. UHPLC separation (2.6 μm, 250 mm × 4.6 mm id., 5 μm on a Thermo UltiMate) 3000 LC system was performed on a COSMOSIL CN column prior to the MS detector. The mobile phase is water eluted with 0.1% formic acid (*v*/*v*) (A) and acetonitrile (B). The elution procedure is as follows: during 0–30 min, A from 5 to 35%, B from 95 to 65%; A from 35 to 100%, B from 65 to 0% in 30–35 min; and A and B remain at 100 and 0% repetitions, respectively, within 35–45 min. The column temperature was maintained at 35°C and eluted at a flow rate of 1 ml/min with an injection volume of 5 μL. A diode array detector (DAD) detects a wavelength of 254 nm and a high-resolution ESI-MS detector is used to record the UHPLC chromatogram. The MS spectrum recorded the system on a Thermo Q-Exactive. Positive and negative mass spectrometers use *m*/*z* 100–1,500 to calibrate the calibration standard mixture of the ionization manufacturer (caffeine, MRFA, and Ultramark 1,621 in acetonitrile-methanol-water solution with 1% acetic acid), allowing mass accuracy in external calibration mode. Not more than 5 ppm, the ionization voltage was 3.5 kV, and the capillary temperature was set to 300°C.

### Establishment of Chemotherapy Treatment Model of H_22_ Hepatocellular Carcinoma Mouse

In aseptic environment, the ascites of mice bearing H_22_ hepatoma for 1 week was extracted and its color was milky white. The number of H_22_ hepatoma cells was counted by inverted microscope (× 100). The concentration of cell suspension was 2 × 10^7^ cells/mL adjusted by normal saline. H_22_ hepatoma cells were subcutaneously inoculated into the right anterior axils of mice at a dose of 0.2 ml/10 g (about 4 × 10^6^/ml). After inoculation of tumor cells for 7 days and tumor mass observed by naked eye, a large dose of 5-FU at 200 mg/kg (0.2 ml/10 g) was injected intraperitoneally to make the model.

### Animal Groupings, Modeling, and Drug Administration

After the chemotherapy model was established, seventy-two mice were randomly divided into six group: model (negative control), 5-FU (20 mg/kg), the YZXJC (0.78 g/kg) treatment, and three CHB-II-F [CHB-II-F (H), CHB-II-F(M), CHB-II-F (L), respectively] treatments. After grouping, the weight of the mice was recorded, and mice were administered 0.2 ml per 10 g body weight. Mice in the model group were given 0.9% physiologic saline for intragastric administration once a day. Mice in 5-FU group were injected intraperitoneally with 5-FU at 20 mg/kg every other day. Mice in YZXJC group were gavaged with 0.039 g/ml YZXJC once a day. CHB-II-F groups received CHB-II-F at the concentrations of 3.25, 1.625, and 0.8125 g/ml [CHB-II-F (H), CHB-II-F (M), or CHB-II-F (L), respectively] by intragastric administration once a day. The treatments continued for 14 days, and mice were fasted for 12 h after the last treatment.

### Measurements of Body Weight and Food Intake

For each cage, mouse food was weighed and recorded every day at 8:00, and the remaining fodder of each cage was weighed and recorded at 8:00 the following day. The difference in food weight was recorded on 2-day basis. The average daily intake of mice was calculated. During the experiment, the mice were weighed and recorded at the same time each day.

### Measurements of Tumor Volume, Weight, and Inhibitory Ratio

The longest diameter (ld) and shortest diameter (sd) of tumor tissue in mice were measured by Vernier caliper on the 7th and 14th days after administration. The tumor volume was calculated according to the formula: V (mm)^3^ = 1/2 × ld (mm) × [sd (mm)]^2^. The mice fasting 12 h after last administration were anesthetized by inhalation of ether. After removing the eyeball to collect the peripheral blood, the mice were killed immediately after dislocating cervical vertebrae. The tumor tissue was removed completely, rinsed with normal saline, drained on the filter paper, and weighed on balance. The tumor inhibitory ratio (IR) was calculated as the average tumor weight of the untreated controls minus the average tumor weight of the treatment group/average tumor weight of untreated controls × 100%.

### Histopathology of Tumor, Stomach, and Duodenum

After the mice were sacrificed, the tumor was stripped off and some of the tumor tissue was cut off. After dissecting the stomach *in vitro* and cutting it off along the great bend of the stomach, the stomach was rinsed with normal saline, and part of the gastric tissue was cut off. The 1-cm duodenum was cut off at the junction of duodenum and gastric antral. Some liver tissues were also removed. The above tissues were fixed in 4% paraformaldehyde for 12 h. The tissues were embedded in paraffin and the 3–5 μm thickness of the slices was cut. All tissues were stained with hematoxylin and eosin and sealed with neutral gum. The pathological changes of the tissues were observed under light microscope and recorded with microscope.

### Content of Serum NPY, Orexin A, Leptin, Ghrelin, GAS, MTL, EGF, PGE2, and Jejunum SOD and MDA Detected by ELISA

After the mice were sacrificed, the jejunum tissue about 3 cm was cut off from the lower end of pylorus and washed with normal saline. After adding appropriate amount of PBS to jejunum tissue, jejunum was homogenized at low temperature. The homogenate was centrifuged at 3,000 rpm for 10 min at a low temperature. The supernatant was collected and stored in the freezer at −80°C. The blood samples were placed at room temperature for 2 h and centrifuged at 3,000 rpm for 10 min at a low temperature. The supernatant was collected and stored in the refrigerator at −80°C. For ELISA, the samples and reagents were left at room temperature for 30 min. The ELISA plate was set up as the standard sample well and sample well; each standard sample well was with different concentration of standard 50 μL. The blank wells and sample wells were tested respectively. The sample diluent 40 μL and the sample 10 μL were added to the sample well on the enzyme-coated plate to make the final dilution of five times. Once the samples were added to the bottom of the enzyme plate wells, they were gently shaken and mixed. In addition to the blank well, the enzyme reagent 100 μL was added to each well. The plate was incubated at 37°C for 60 min. After carefully removing the sealing membrane, discarding the liquid, and spinning dry, each well was filled with washing fluid, rested for 30 s, and discarded for five times. The 50 μL chromogenic agent A was added to each well, and then B 50 μL was added in each hole. The agency was mixed gently and incubated at 37°C for 15 min. The reaction was terminated by adding 50 μL of the termination solution to each well. The blank well is set to zero, and the 450 nm wavelength is used to measure the absorbance of each well in order (OD value). The determination should be carried out within the 15 min after the termination solution is added. The results were repeated three times.

### NPY, AgRP, POMC, CART, OX1R, Ob-R, and GHSR Protein Expressed in Hypothalamus Detected by Western Blot

After the blood was immediately taken, the mice were sacrificed by dislocated cervical vertebrae. The hypothalamus of the mice was quickly removed on the sterile ice and stored in the freezer at −80°C. The total protein was extracted from the hypothalamus of mice in each group and quantified by the BCA protein quantitative kit (Multi Sciences, China). The total protein from each group was loaded onto each well of 12 or 15% SDS-PAGE gel according to the molecular weight of the protein and separated by electrophoresis. The protein on SDS-PAGE gel was transferred to PVDF membrane by electroporation. After electroporation, PVDF was immersed in a sealed solution containing 5% skimmed milk powder in PBST and incubated at room temperature for 1 hour. After washing three times for 5 min each time, NPY antibody (1:1,000), AgRP antibody (1:1,000), POMC antibody (1:1,000), CART antibody (1:1,000), OX1R antibody (1:500), Ob-R antibody (1:1,000), GHSR antibody (1:500), and GAPDH antibody (1:5,000) were added, respectively, and incubated overnight at 4°C. After washing with PBST three times with 20 min each time, the horseradish peroxidase labeled anti-di antibody (Proteintech, United States) was added and incubated at room temperature for 1 h. After washing with PBST three times of 10 min each time, the PVDF film was incubated with freshly prepared ECL chemiluminescent solution (Beyotime, China) for 2 min in the dark room. The optical density of target strip and GAPDH internal reference strip were analyzed by ImageJ image analyzer software.

### NPY, AgRP, POMC, CART, OX1R, Ob-R, and GHSR mRNA Expression in Hypothalamus Detected by RTFQ-PCR

The concentration of total RNA extracted from hypothalamus by Trizol extraction kit was adjusted to 0.5 μg/μL for all mRNA samples. Using 1% agarose gel for electrophoresis, the electrophoresis bands of RNA were observed under the gel imaging system. The ribosomal RNA (rRNA) 28 and 18 s bands were compared to determine their integrity. Reverse transcription was performed according to the instructions of the first strand cDNA synthesis kit. Real-time fluorescence quantitative PCR (RTFQ-PCR) amplification was carried out in 25 μL reaction system. The primers were designed with the software Primer Premier 5.0. The sequence of primers was as follows: β-actin, forward 5′-CTGT CCCT GTAT GCCT CTG-3′, reverse 5′-ATGT CACG CACG ATTT CC-3′, product length 219bp; NPY, forward 5′-TAGG TAAC AAGC GAAT GGG-3′, reverse 5′-TGAT GTAG TGTC GCAG AGC-3′, product length 213bp; AgRP, forward 5′-CAGA CCGA GCAG AAGA AGTT-3′, reverse 5′-CATT GAAG AAGC GGCA GTA-3′, product length 177bp; POMC, forward 5′-TGCT GCTG GTCT TGCT GCTT-3′,reverse 5′-CCGT CTCC TCCT CACG CTTC T-3′, product length 156bp; CART, forward 5′-TCTG CCGT GGAT GATG CG-3′, reverse 5′-GGAA TGCG TTTA CTCT TGAG C-3′, product length 257bp; Ob-R, forward 5′-GTGT AAAC TGGG ACAT AGAG-3′, reverse 5′-CAGA AGAG CGTA GTTG AGT-3′, product length 231bp; GHSR, forward 5′-GCAC GAGA ACGG CACA GA-3′, reverse 5′-CGGC AGGA AGAA GAAG ACG-3′, product length 118bp; OX1R, forward 5′-GGTG CGGA ACTG GAAA CG-3′, reverse 5′-CTGG CTTG GCGG AACA TC-3′, product length 125bp. The amplification conditions were as follows: predenaturation at 95°C for 3 min, denaturation at 94°C for 30 s, annealing temperature at 56°C for 30 s, and extension at 72°C for 30 s for 35 cycles. 2^−ΔΔct^ was used to analyze the results of gene expression, and β-actin expression was used as internal reference. The results were repeated 3 times.

### Expression of NPY, AgRP, POMC, CART, OX1R, Ob-R, and GHSR Protein in Hypothalamus as well as 5-HT and Substance P Protein in Duodenum Detected by Immunohistochemistry

The hypothalamic and duodenal tissues were embedded in paraffin, sliced and dewaxed. The slices were soaked in different concentrations of ethanol for gradient dehydration and washed in ddH_2_O with 5 min for 3 times. After antigen repair, the slides were washed with ddH_2_O twice. After washing with immunostaining solution and marking with immunohistochemical oily pen to circle the tissue sample position, the slides were added with 3% H_2_O_2_-methanol. After washing, the slides were sealed by immunostaining solutions for 45 min. The dilutions of rabbit anti-Orexin receptor 1 antibody, rabbit anti-Leptin Receptor antibody, rabbit anti-Ghrelin receptor antibody, rabbit anti-substance P antibody, and rabbit anti-5-HT antibody were 1:400; POMC antibody was 1: 100; and NPY antibody, AgRP antibody, and CART antibody were 1:50. The slides were incubated with the first antibody diluents overnight in refrigerator at 4°C. The next day, the slices were placed at room temperature for 60 min. After washing, HRP-conjugated antibody was added and incubated for 45 min at room temperature. After washing again, the prepared DAB chromogenic solution was added to the slides and stained in the wet box. The color reaction was terminated with distilled water. After restaining with hematoxylin and decolorizing to blue, the slides were sealed with neutral resin and observed under microscope.

The expression of protein was positive in the granular brown or brown part of the sections. Image-Pro Plus 6.0 image analysis software was used for quantitative statistical analysis of each slide. Five visual fields (× 400) were randomly selected from each slide for statistical analysis. The cumulative optical density value (IOD value) of the positive expression site in each slide was measured. The higher the IOD value, the stronger the positive expression.

### Statistical Analysis

The data were expressed as mean ± SD ($\bar{x}$ ± *s*). Graph Pad Prism 7.0 software (GraphPad Software Inc., La Jolla, United States) was used for statistical analysis. Normal distribution and homogeneity test between groups were performed. Statistical significance was determined by using one-way analysis of variance (One-Way ANOVA), followed by the least significant difference (LSD) post hoc test. The Kruskal-Wallis nonparametric H test was used when there was one inconsistency. Differences with *p* < 0.05 were considered significant.

## Results

### Identification of Compounds in CHB-II-F

**FIGURE 1:**
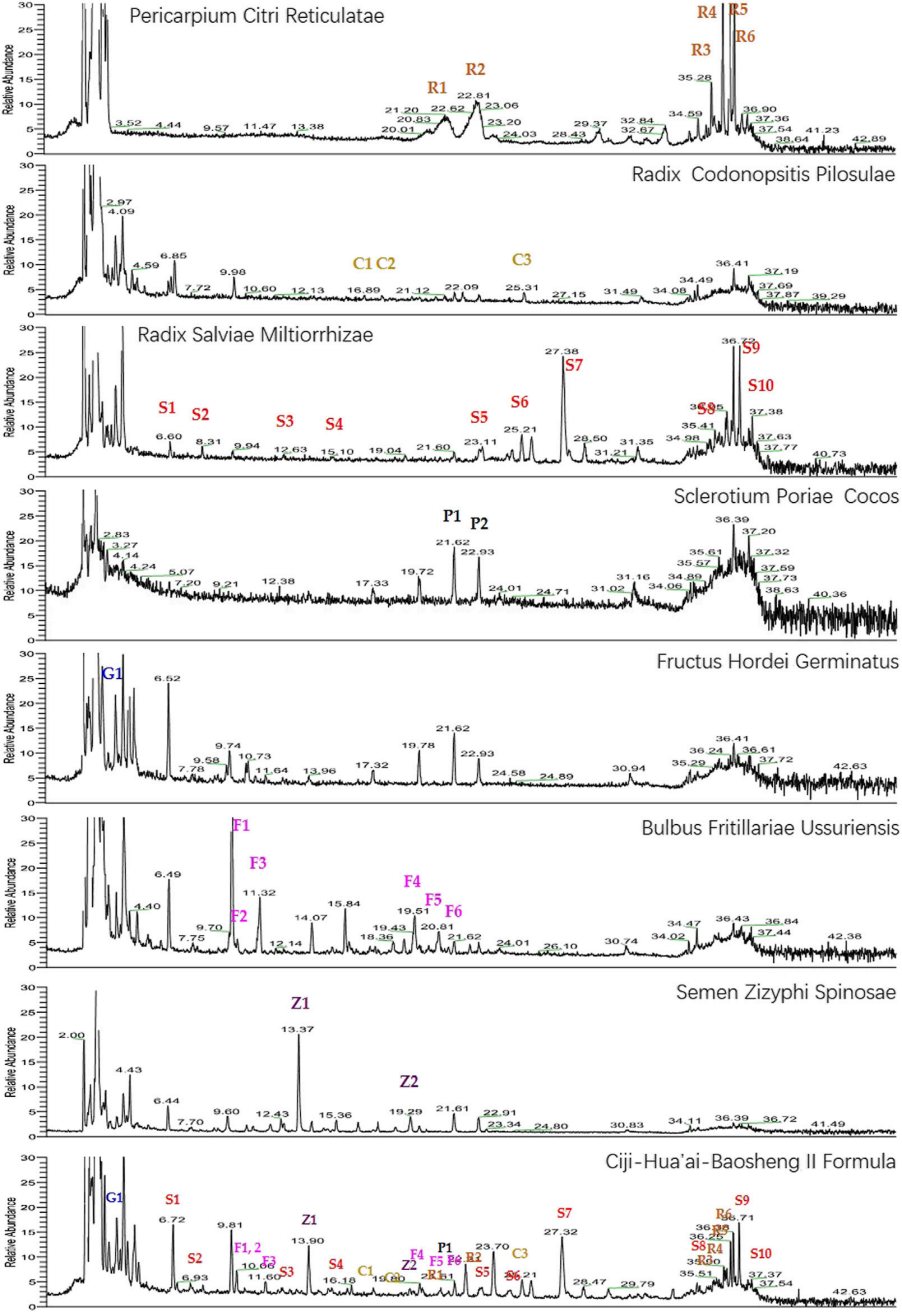
UHPLC-MS chemical fingerprint chromatogram of CHB-II-F and each herb. Naringin (R1), hesperidin (R2), 5,7,8,4′-tetramethoxyflavone (R3), nobiletin (R4), 3,5,6,7,8,3′,4′-heptamethoxyflavone (R5), tangeretin (R6), phenylalanine (S1), salvianolic aid A (S2), protocatechualdehyde (S3), caffeic acid (S4), lithospermic acid (S5), rosmarinic acid (S6), salvianolic acid B (S7), tanshinone IIA (S8), cryptotanshinone (S9), dihydrotanshinone I (S10), tangshenoside I (C1), tangshenoside II (C2), kukoamine B (C3), thalictrine (Z1), spinosin (Z2), nicotinic acid (G1), pingpeimine B (F1), pingpeimine A (F2), pingpeimine C (F3), peimisine (F4), peimine (F5), and peiminine (F6).

As shown in Figure 1, the chromatographic fingerprints of CHB-II-F and each herb were constructed. The 28 peaks were identified by comparing their UV and MS spectra with published data or standard compounds (Figure 1, Supplementary Table S1): naringin (R1), hesperidin (R2), 5,7,8,4′-tetramethoxyflavone (R3), nobiletin (R4), 3,5,6,7,8,3′,4′-heptamethoxyflavone (R5), tangeretin (R6), phenylalanine (S1), salvianolic aid A (S2), protocatechualdehyde (S3), caffeic acid (S4), lithospermic acid (S5), rosmarinic acid (S6), salvianolic acid B (S7), tanshinone IIA (S8), cryptotanshinone (S9), dihydrotanshinone I (S10), tangshenoside I (C1), tangshenoside II (C2), kukoamine B (C3), thalictrine (Z1), spinosin (Z2), nicotinic acid (G1), pingpeimine B (F1), pingpeimine A (F2), pingpeimine C (F3), peimisine (F4), peimine (F5), and peiminine (F6).

### Effects of CHB-II-F on Body Weight and Food Intake

**FIGURE 2:**
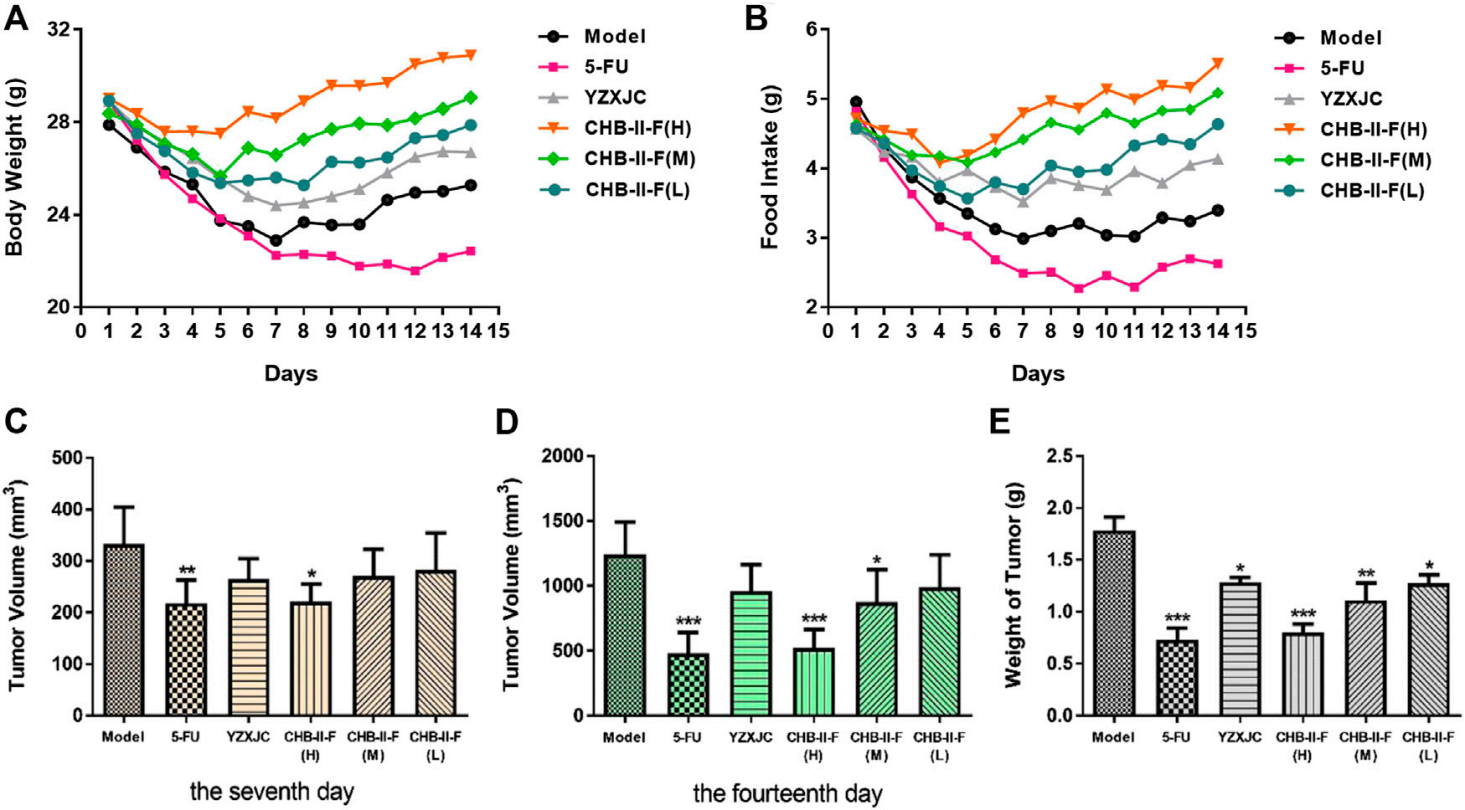
Effects of CHB-II-F on body weight, intake, tumor volume, and weight of mice with transplanted H_22_ hepatocellular carcinoma after chemotherapy ($\bar{x}$±*s*, *n* = 6–8). Statistical analysis: *p < 0.05, **p < 0.01, ***p < 0.001 compared with the model (saline) group.

After 14 days of continuous treatment, compared with the untreated group and the 5-FU group, the weight and intake of the mice decreased slightly in all the CHB-II-F-treated groups and the YZXJC group, and the body weight and food intake were recovered quicker. Compared with YZXJC group, the decreases of body weight and food intake in all the CHB-II-F-treated groups were smaller, and the recoveries of body weight and food intake were faster, and there was a dose-dependent manner of CHB-II-F in three CHB-II-F-treated groups (Figure 2).

### Effects of CHB-II-F on Tumor Weight, Volume, and Tumor Inhibition Ratio

Compared with the untreated group, on the 7th day after treatment, the tumor volume of the 5-FU group and CHB-II-F(H) group decreased significantly (*p* < 0.05 or *p* < 0.01). On the 14th day after the treatment, the tumor volume of 5-FU group, CHB-II-F(H) and CHB-II-F(M) groups decreased significantly, and there was statistical significance (*p* < 0.05 or *p* < 0.001). Compared with the untreated group, the tumor weights of all the CHB-II-F-treated groups, 5-FU group, and YZXJC group were significantly smaller (*p* < 0.05, *p* < 0.01, or *p* < 0.001) (Figure 2). The tumor inhibition ratios (IRs) of 5-FU group, YZXJC group, CHB-II-F(H), CHB-II-F(M), and CHB-II-F(L) were 58.88, 28.08, 54.96, 37.69, and 28.61%, respectively.

### Effects of CHB-II-F on Pathology of Tumor, Stomach, Duodenum, and Liver

**FIGURE 3:**
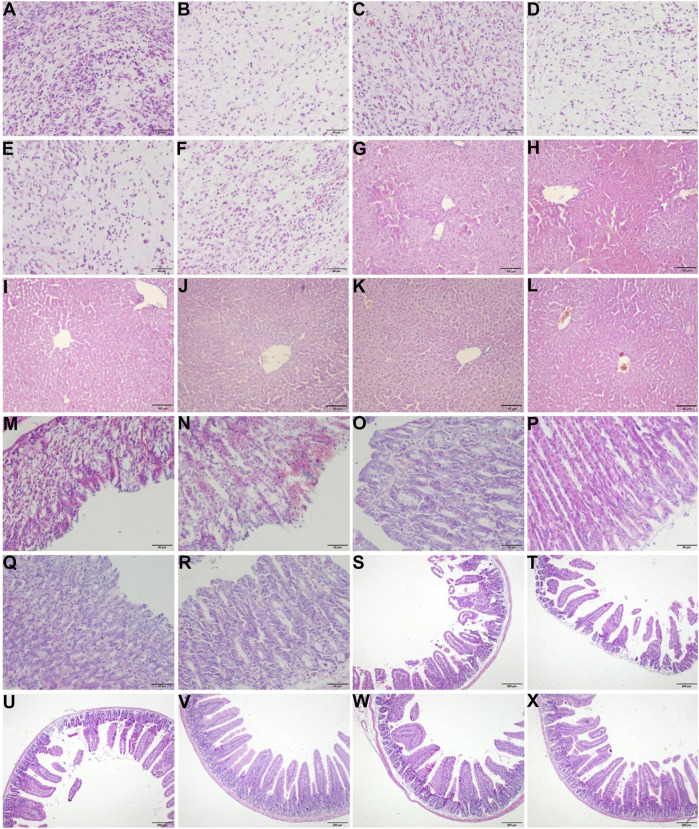
Effects of CHB-II-F on pathology of tumor, liver, gastric, and duodenal tissues in the transplanted H_22_ hepatocellular carcinoma model mice receiving chemotherapy. Sections were stained with hematoxylin and eosin (H and E) and viewed at a magnification of × 400 [for tumor **(A–F)** and gastric tissue **(M–R)**], × 200 [for liver tissue **(G–L)**], or × 100 [for duodenal tissue **(S–X)**; **(A) (G) (M) (S)**. Model (saline/negative control); **(B) (H) (N) (T)**. 5-FU-treated control (20 mg/kg); **(C) (I) (O) (U)**. Yangzheng Xiaoji capsule-treated control (0.78 g/kg); **(D) (J) (P) (V)**. CHB-II-F (65 g/kg); **(E) (K) (Q) (W)**. CHB-II-F (32.5 g/kg); **(F) (L) (R) (X)**. CHB-II-F (16.25 g/kg). Scale bar, 50 μm for **(A–R)** and 200 μm for **(S–X)**.

As shown in Figures 3A–F, the tumor cells in the untreated group were dense, scattered, different in size and shape, and highly heteromorphic. The most tumor cells were dark in color, pink in cytoplasm, and large in nuclear volume after staining. In the 5-FU group, the staining of tumor cells was lighter, the number of cells decreased obviously, the arrangement of cells was looser, and the nucleus began to shrink and rupture. Compared with the untreated and YZXJC groups, the number of tumor cells in CHB-II-F-treated groups decreased significantly, the arrangement of the cells was looser, the mitosis was decreased, and the heteromorphism was lower.

As shown in Figures 3M–R, the epithelial structure of gastric mucosa in the untreated group was incomplete, the gastric fundus glands were scattered and irregular, and a large number of blood cells accumulated in the epithelial structure. In 5-FU group, the epithelial structure of gastric mucosa was incomplete, the gastric fundus glands were scattered and irregular, and even there were appearance of shedding and deletion. A large number of blood cells accumulated in the epithelial structure, and inflammatory cells infiltrated into the lamina propria. In YZXJC group, the epithelial structure of gastric mucosa was disordered, inflammatory cells were infiltrated, and scattered blood cells were still distributed in the epithelial structure. In CHB-II-F-treated groups, the epithelial structure of gastric mucosa was intact, the glands were well distributed, the gastric fundus glands in the lamina propria were closely arranged, and there was no aggregation of blood cells and no infiltration of inflammatory cells.

As shown in Figures 3S–X, a large number of intestinal villi in the untreated group were damaged, and the intestinal villi in the 5-FU group were shortened or shedded, which showed atrophy, loss, severe injury, and disorder of intestinal villi arrangement. There was a reduction of intestinal villi breakage and deletion in YZXJC group. In the groups of high, middle, and low dose of CHB-II-F, the structure of small intestinal mucosa was intact, the glands were arranged in order, and some villi were slightly damaged.

As shown in Figures 3G–L, HE staining of liver showed almost normal hepatic structure. The hepatic lobule structures were clear and intact. Necrosis of hepatocytes and infiltration of any inflammatory cells were not found. And other obvious lesions were also not found.

### Effects of CHB-II-F on Content of Serum NPY, Orexin A, Leptin, Ghrelin, GAS, MTL, EGF, PGE2, and Jejunum SOD and MDA

**FIGURE 4:**
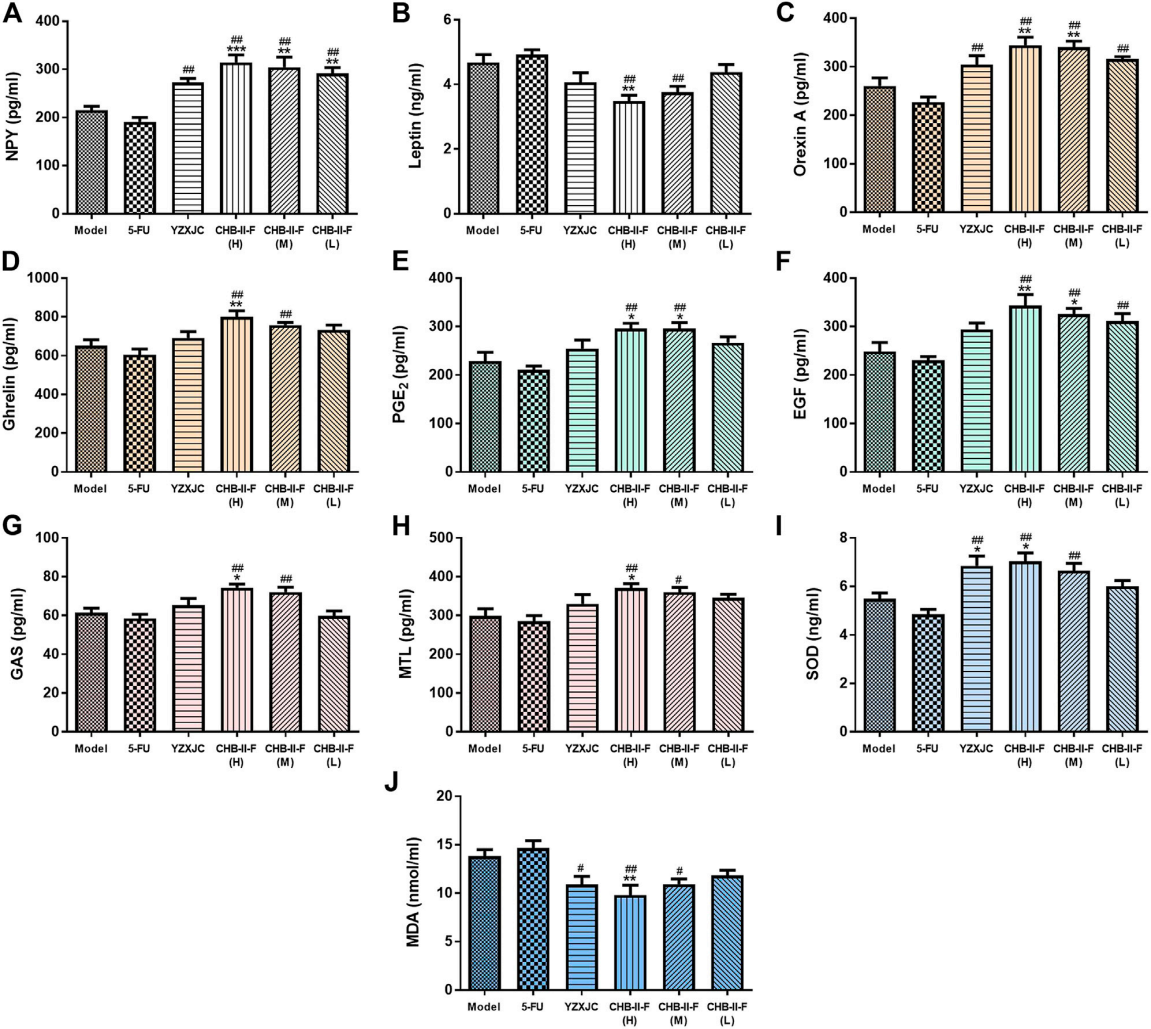
Effects of CHB-II-F on the content of NPY, OX1R, Ghrelin, Leptin, GAS, MTL, PGE_2_, and EGF in serum, SOD, and MDA in the jejunum of the transplanted H_22_ hepatocellular carcinoma model mice receiving chemotherapy ($\bar{x}$ ± *s*, *n* = 6–8). Statistical analysis: *p < 0.05, **p < 0.01, ***p < 0.001 compared with the model (saline) group. ^#^ *p* < 0.05, ^##^ *p* < 0.01 compared with the 5-FU group.

The results of ELISA showed that, compared with the untreated group, the contents of PGE_2_, Orexin A, EGF, and NPY in CHB-II-F(H) and CHB-II-F(M) groups were significantly higher (*p* < 0.05, *p* < 0.01, or *p* < 0.001). The NPY content in CHB-II-F(L) group and the Ghrelin content in CHB-II-F(H) were significantly higher (*p* < 0.01). The contents of GAS and MTL in CHB-II-F(H) group were also significantly increased (*p* < 0.05). Compared with 5-FU group, the contents of NPY, Orexin A in YZXJC group and CHB-II-F groups were significantly increased (*p* < 0.01). The contents of EGF, Ghrelin, PGE_2_, GAS, MTL in CHB-II-F(H) and CHB-II-F(M) groups were significantly higher (*p* < 0.05 or *p* < 0.01). The content of EGF in CHB-II-F(L) was also significantly increased (*p* < 0.01). Compared with the untreated group, the Leptin content in CHB-II-F(H) group was significantly lower (*p* < 0.01). Compared with the 5-FU group, the content of Leptin in CHB-II-F(H) and CHB-II-F(M) groups was decreased (*p* < 0.01) (Figures 4A–H).

Compared with the untreated group, the activity of SOD in CHB-II-F(H) and YZXJC group was significantly increased (*p* < 0.05). The content of MDA in CHB-II-F(H) group was significantly decreased (*p* < 0.01). Compared with 5-FU group, the activity of SOD in YZXJC, CHB-II-F(H) and CHB-II-F(M) groups was increased (*p* < 0.01), and the content of MDA was significantly decreased (*p* < 0.01 or *p* < 0.05) (Figures 4I,J).

### Effects of CHB-II-F on Protein Expression of NPY, AgRP, POMC, CART, OX1R, Ob-R, and GHSR in Hypothalamus

**FIGURE 5:**
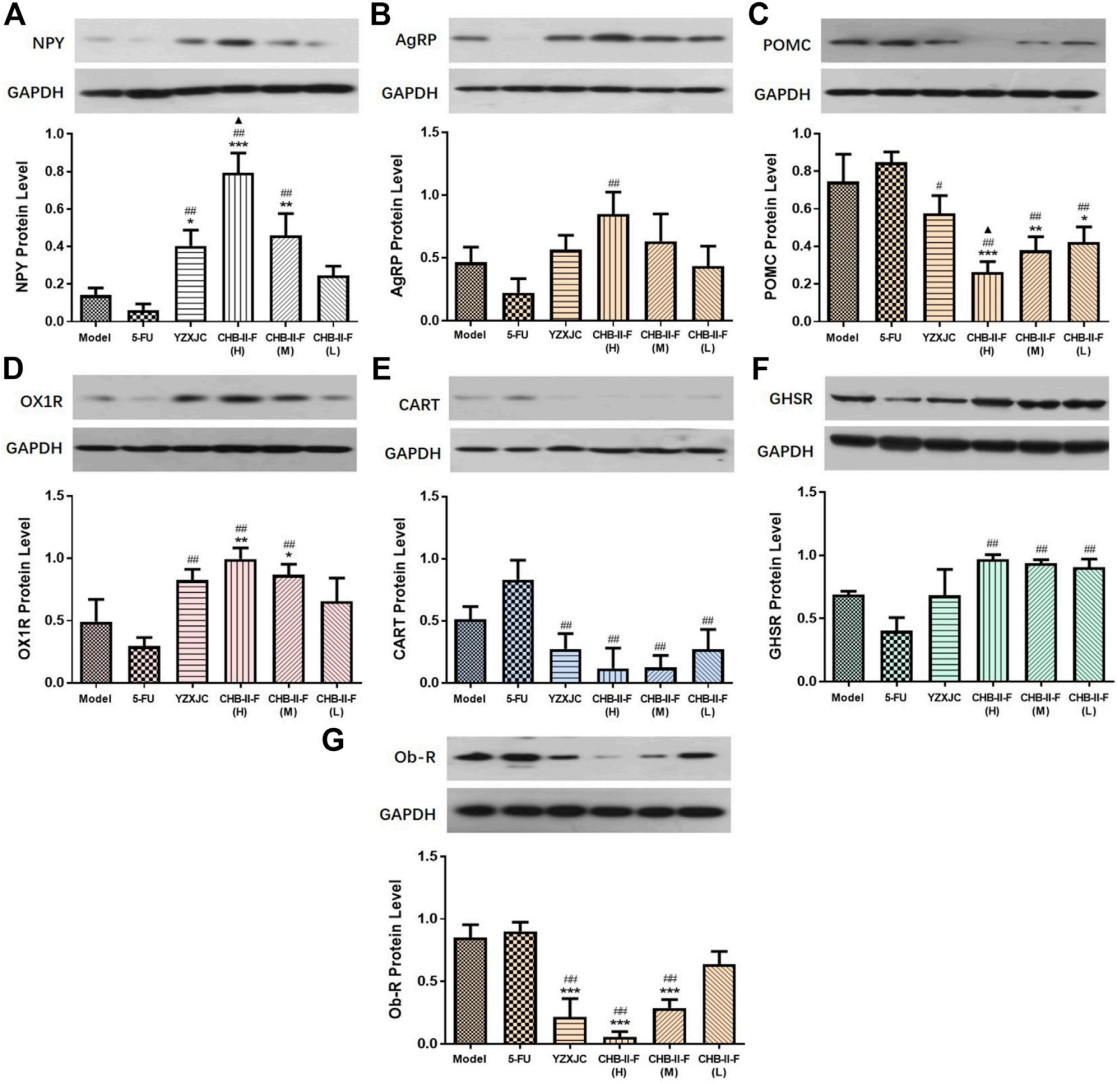
Effects of CHB-II-F on protein expression of NPY, AgRP, POMC, CART, OX1R, Ob-R, and GHSR in the hypothalamus of the transplanted H_22_ hepatocellular carcinoma model mice receiving chemotherapy ($\bar{x}$±*s*, n = 6). Statistical analysis: *p < 0.05, **p < 0.01, ***p < 0.001 compared with the model (saline) group. ^#^ *p* < 0.05, ^##^ *p* < 0.01 compared with the 5-FU group, ^▲^ *p* < 0.05 compared with the YZXJC group.

As shown in Figure 5, compared with the untreated group, the protein expression of NPY and OX1R in CHB-II-F(H) and CHB-II-F(M) groups were significantly increased (*p* < 0.05, *p* < 0.01, or *p* < 0.001), and the level of NPY in YZXJC group was significantly higher (*p* < 0.05). Compared with 5-FU group, the protein expression of NPY, AgRP, GHSR, and OX1R in CHB-II-F(H) group was significantly higher (*p* < 0.01). The protein expression of NPY, GHSR, and OX1R was significantly increased in CHB-II-F(M) group (*p* < 0.01). The protein expression of GHSR in CHB-II-F(L) group was significantly higher (*p* < 0.01), and the expression of NPYand OX1R protein in YZXJC group was significantly increased (*p* < 0.01). Compared with the YZXJC group, the expression of NPYt protein in the CHB-II-F(H) group was significantly higher (*p* < 0.05). Compared with the untreated group, the protein expression of POMC in all the CHB-II-F-treated groups was significantly lower (*p* < 0.05, *p* < 0.01, or *p* < 0.001), and the expression of Ob-R protein in YZXJC, CHB-II-F(H), and CHB-II-F(M) groups was significantly decreased (*p* < 0.001). Compared with 5-FU group, the protein expression of POMC and CART in all the CHB-II-F-treated groups and YZXJC group was significantly decreased (*p* < 0.05 or *p* < 0.01), and the expression of Ob-R protein in YZXJC, CHB-II-F(H), and CHB-II-F(M) groups was significantly lower (*p* < 0.01). Compared with the YZXJC group, the expression of POMC protein in CHB-II-F(H) was significantly decreased (*p* < 0.05).

### Effects of CHB-II-F on mRNA Expression of NPY, AgRP, POMC, CART, OX1R, Ob-R, and GHSR in Hypothalamus

**FIGURE 6:**
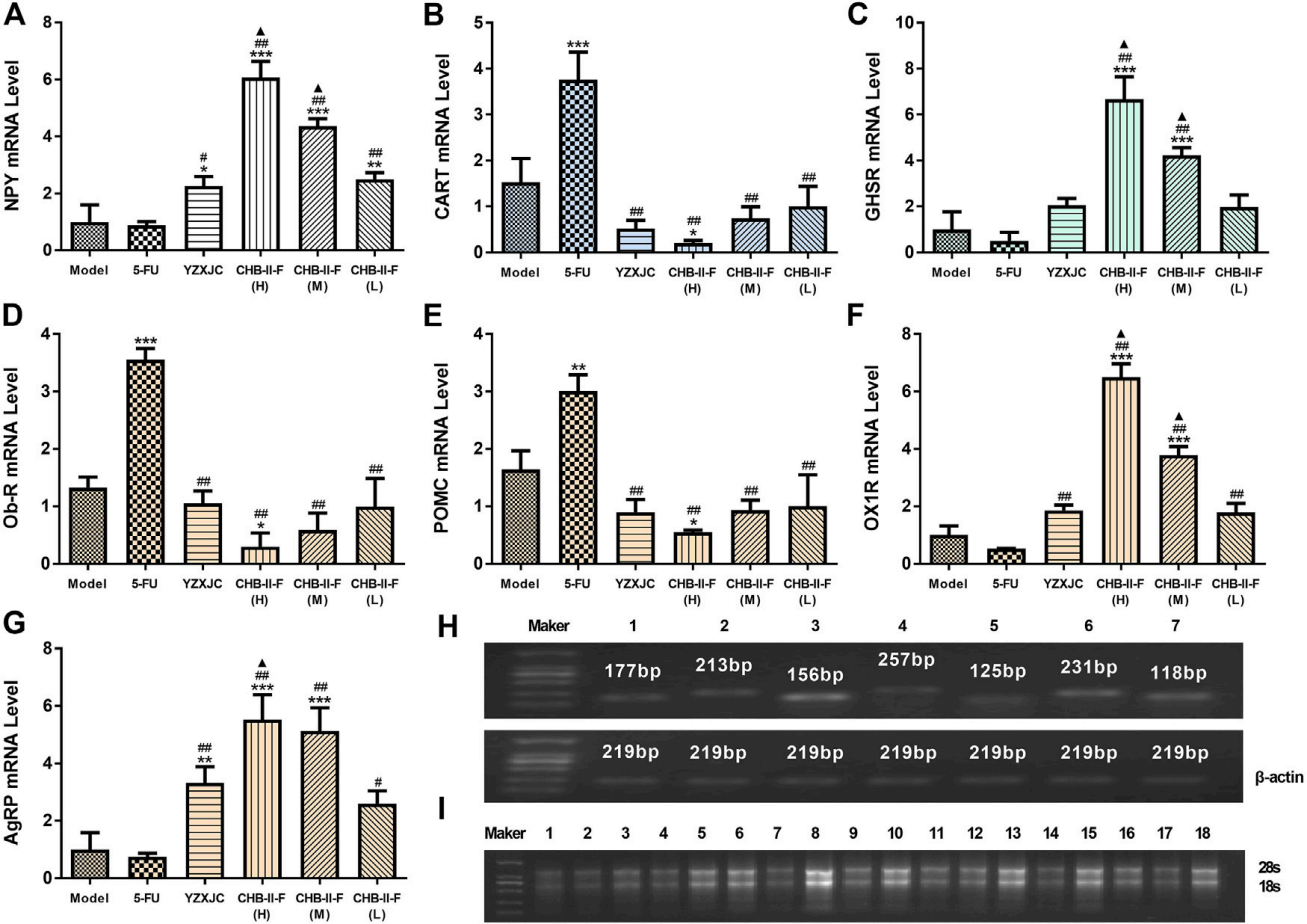
Effects of CHB-II-F on mRNA expression of NPY, AgRP, POMC, CART, OX1R, Ob-R,., and GHSR in the hypothalamus of the transplanted H_22_ hepatocellular carcinoma model mice receiving chemotherapy ($\bar{x}$ ± *s*, *n* = 6). **(A)** NPY mRNA expression in different groups. **(B)** CART mRNA expression in different groups. **(C)** GHSR mRNA expression in different groups. **(D)** Ob-R mRNA expression in different groups. **(E)** POMC mRNA expression in different groups. **(F)** OX1R mRNA expression in different groups. **(G)** AgRP mRNA expression in different groups. **(H)** Gel electrophotogram of target gene NPY, AgRP, POMC, CART, OX1R, Ob-R, GHSR, and β-actin amplification products of real-time fluorescent quantitation polymerase chain reaction (RTFQ-PCR). 1: AgRP; 2: NPY; 3: POMC; 4: CART; 5: OX1R; 6: Ob-R; 7: GHSR. **(I)** Gel electrophotogram of total RNA of tumor tissue. Randomly select three samples from each group and verify their integrity. 1–3: saline-treated controls (model); 4–6: 5-FU treated controls (0.02 g/kg); 7–9: YZXJC (0.78 g/kg); 10–12: CHB-II-F(H) (65 g/kg); 13–15: CHB-II-F(M) (32.5 g/kg); 16–18: CHB-II-F(L) (16.25 g/kg). Statistical analysis: *p < 0.05, **p < 0.01, ***p < 0.001 compared with the model (saline) group. ^#^ *p* < 0.05, ^##^ *p* < 0.01 compared with the 5-FU group. ^▲^ *p* < 0.05 compared with the YZXJC group.

Compared with the untreated group, the gene expression of NPY in all the CHB-II-F-treated groups and YZXJC group was significantly higher (*p* < 0.05, *p* < 0.01, or *p* < 0.001), the AgRP gene expression of YZXJC, CHB-II-F(H), and CHB-II-F(M) groups was significantly increased (*p* < 0.01 or *p* < 0.001), and the OX1R and GHSR gene expression was significantly increased in CHB-II-F(H) and CHB-II-F (M) groups (*p* < 0.001). Compared with 5-FU group, the gene expression of NPY, AgRP, and OX1R in all the CHB-II-F-treated groups and YZXJC group was significantly higher (*p* < 0.05 or *p* < 0.01), and the gene expression of GHSR in CHB-II-F(H) and CHB-II-F (M) groups was significantly increased (*p* < 0.01). Compared with the YZXJC group, the expression of NPY, OX1R, and GHSR genes in CHB-II-F(H) and CHB-II-F (M) groups was significantly increased (*p* < 0.05), and the gene expression of AgRP in CHB-II-F(H) group was significantly higher (*p* < 0.05). Compared with the untreated group, the expression of POMC, CART, and Ob-R genes in CHB-II-F(H) group was significantly lower (*p* < 0.05). Compared with the 5-FU group, the gene expression of CART, Ob-R, and POMC in all the CHB-II-F-treated groups and YZXJC group was significantly decreased (*p* < 0.01) (Figure 6).

### Effects of CHB-II-F on Expression of NPY, AgRP, POMC, CART, OX1R, Ob-R, and GHSR Protein in Hypothalamus by Immunohistochemistry

**FIGURE 7:**
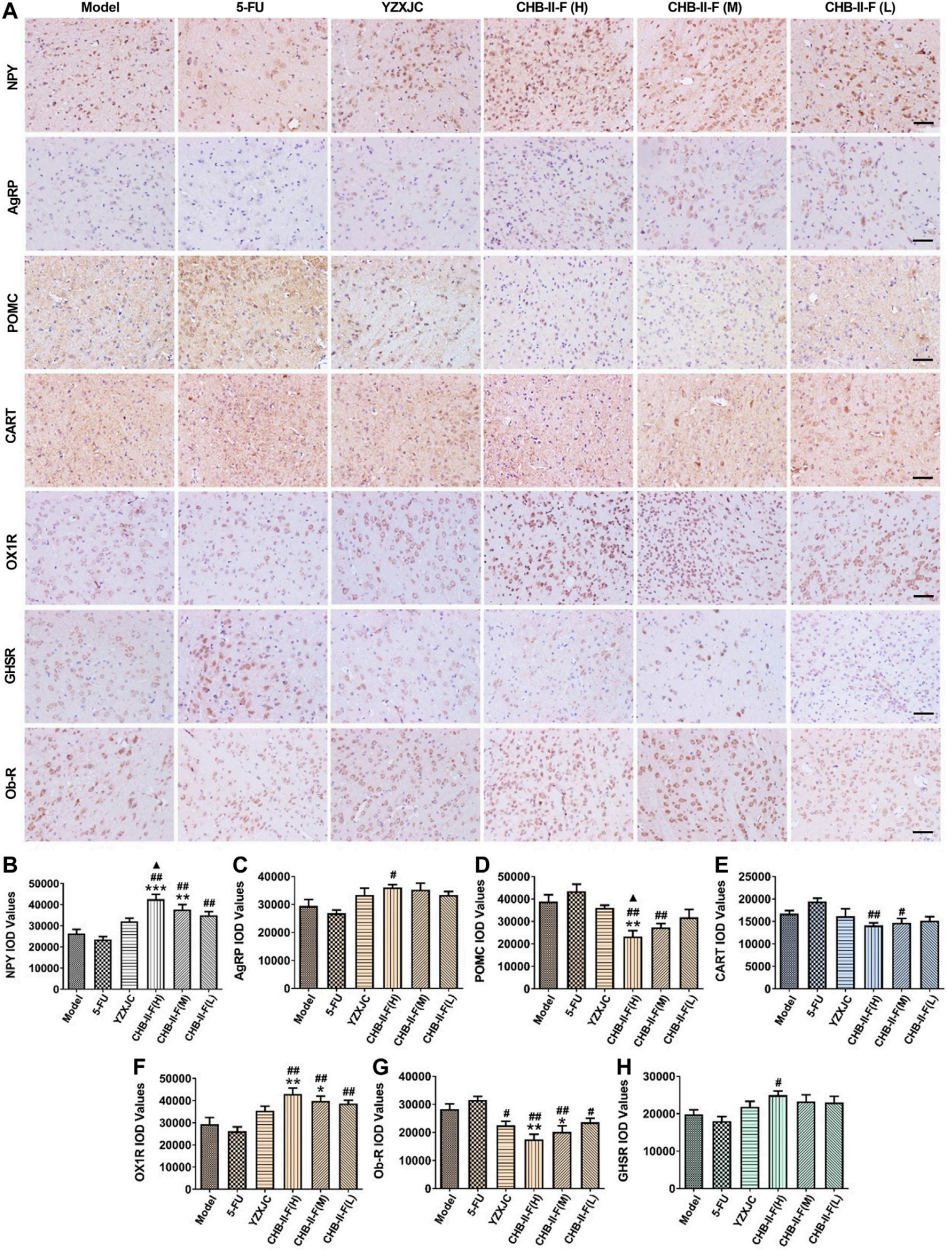
Effects of CHB-II-F on protein expression of NPY, AgRP, POMC, CART, OX1R, Ob-R, and GHSR in the hypothalamus of the transplanted H_22_ hepatocellular carcinoma model mice receiving chemotherapy detected by immunohistochemistry ($\bar{x}$ ± *s*, *n* = 6). **(A)** Immunohistochemical pictures of NPY, AgRP, POMC, CART, OX1R, Ob-R, and GHSR protein expression in hypothalamus (× 400, scale bar = 200 μm). **(B)** NPY IOD values in different groups. **(C)** AgRP IOD values in different groups. **(D)** POMC IOD values in different groups. **(E)** CART IOD values in different groups. **(F)** OX1R IOD values in different groups. **(G)** Ob-R IOD values in different groups. **(H)** GHSR IOD values in different groups. Statistical analysis: *p < 0.05, **p < 0.01, ***p < 0.001 compared with the model (saline) group. ^#^ *p* < 0.05, ^##^ *p* < 0.01 compared with the 5-FU group. ^▲^ *p* < 0.05 compared with the YZXJC group.

The protein expression of NPY and OX1R in CHB-II-F(H) and CHB-II-F(M) groups was significantly higher than that in the untreated group (*p* < 0.05, *p* < 0.001, or *p* < 0.001). Compared with 5-FU group, the protein expression of NPY and OX1R in high-, middle-, and low-dose groups of CHB- Ⅱ-F was significantly increased (*p* < 0.01), and the expression of AgRP and GHSR in the high-dose group of CHB-Ⅱ-F was significantly increased (*p* < 0.05). Compared with YZXJC group, the expression of NPY protein in CHB-Ⅱ-F(H) group was significantly increased, which was statistically significant (*p* < 0.05) (Figure 7).

The expression of POMC and Ob-R protein in the high-dose group of CHB-Ⅱ-F was significantly lower than that in the untreated group (*p* < 0.01), and the expression of Ob-R protein in the middle-dose group of CHB-Ⅱ-F was significantly lower than that in the untreated group (*p* < 0.05). Compared with 5-FU group, the expression of CART, POMC, and Ob-R protein in CHB-II-F(H) and CHB-II-F(M) groups was significantly lower than that in 5-FU group, and the expression of Ob-R protein in CHB-Ⅱ-F(L) group and YZXJC group was significantly lower than that in YZXJC group (*p* < 0.05). Compared with YZXJC group, the expression of POMC protein in CHB-Ⅱ-F(H) group was significantly decreased (*p* < 0.05) (Figure 7).

### Effects of CHB-II-F on Expression of 5-HT and Substance P Protein in Duodenum by Immunohistochemistry

**FIGURE 8:**
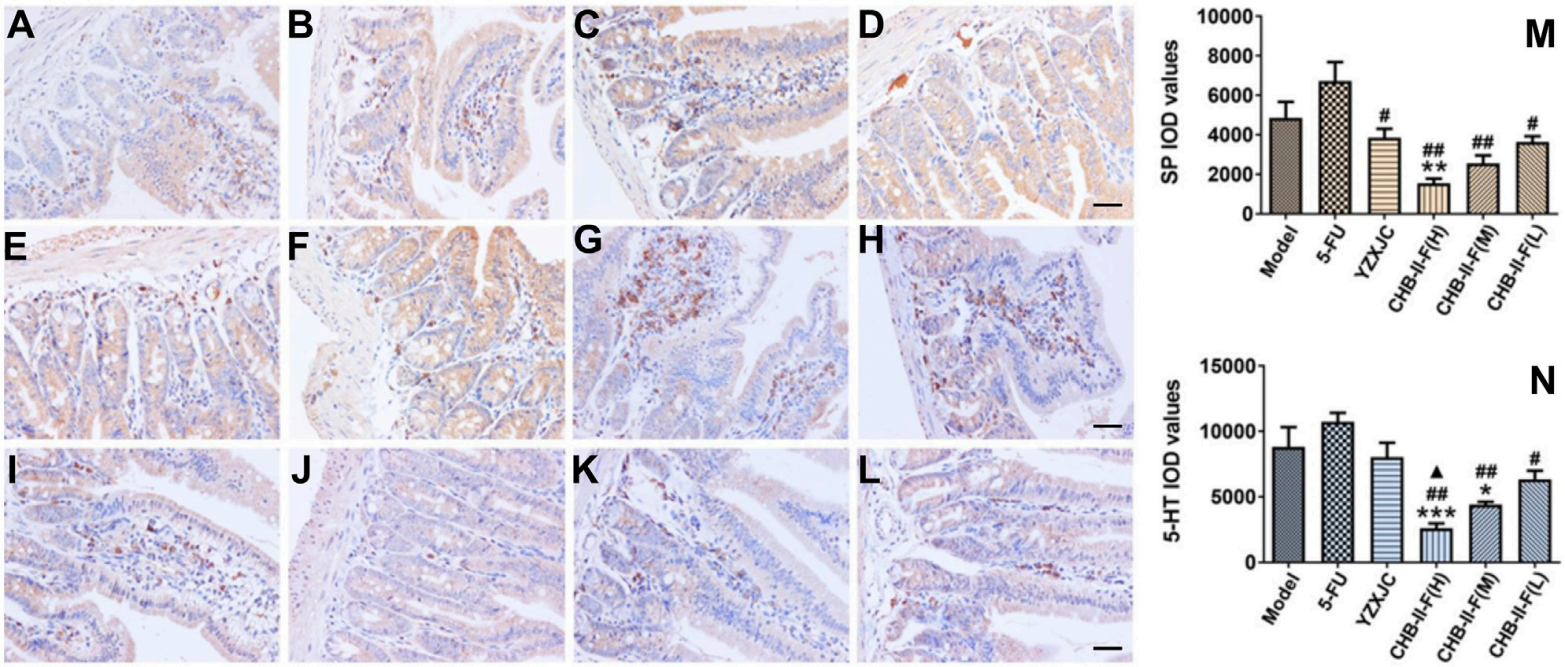
Effects of CHB-II-F on protein expression of SP and 5-HT in the duodenum of the transplanted H_22_ hepatocellular carcinoma model mice receiving chemotherapy detected by immunohistochemistry ($\bar{x}$±*s*, *n* = 6). **(A–F)** Immunohistochemical pictures of SP protein expression in duodenum in saline-treated controls (model), 5-FU treated controls (0.02 g/kg), YZXJC (0.78 g/kg), CHB-II-F(H) (65 g/kg), CHB-II-F(M) (32.5 g/kg), and CHB-II-F(L) (16.25 g/kg), respectively (× 400, scale bar = 200 μm). **(G–L)** Immunohistochemical pictures of 5-HT protein expression in duodenum in saline-treated controls (model), 5-FU treated controls (0.02 g/kg), YZXJC (0.78 g/kg), CHB-II-F(H) (65 g/kg), CHB-II-F(M) (32.5 g/kg), and CHB-II-F(L) (16.25 g/kg), respectively (× 400, scale bar = 200 μm). **(M)** SP IOD values in different groups. **(N)** 5-HT IOD values in different groups. Statistical analysis: *p < 0.05, **p < 0.01, ***p < 0.001 compared with the model (saline) group. ^#^ *p* < 0.05, ^##^ *p* < 0.01 compared with the 5-FU group. ^▲^ *p* < 0.05 compared with the YZXJC group.

Compared with the untreated group, the expression of 5-HT and substance P (SP) protein in CHB-II-F(H) group was significantly decreased (*p* < 0.001 or *p* < 0.01), and the 5-HT protein expression in CHB-II-F(M) group was also decreased (*p* < 0.05). Compared with 5-FU group, the protein expression of 5-HT and SP in CHB-II-F(H), CHB-II-F(M), and CHB-II-F(L) groups was significantly decreased (*p* < 0.05 or *p* < 0.01), and the expression of SP protein in YZXJC group was significantly lower (*p* < 0.05). Compared with YZXJC group, the expression of 5-HT protein decreased significantly in CHB-II-F(H) group (*p* < 0.05) (Figure 8).

## Discussion

**FIGURE 9:**
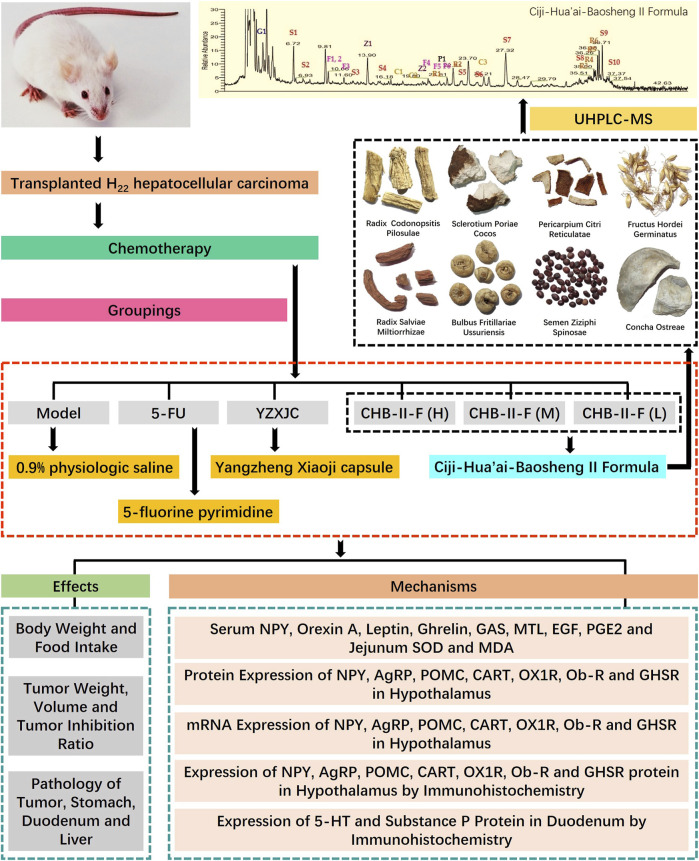
Research conducted in this study. This study was divided into three main parts: identification of chief components in CHB-II-F using UHPLC-MS; verification of the therapeutic effects of CHB-II-F in chemotherapy-treated mice; and exploration of the mechanism underlying CHB-II-F using ELISA, western blot, RTFQ-PCR, and IHC to detect appetite-related factors.

Malignant tumor is still one of the most important causes of human death, and chemotherapy is one of the most common methods in clinical treatment of cancer. Although chemotherapy can inhibit or even kill tumor cells to a certain extent, its severe toxicity to gastrointestinal tract seriously limits its extensive application in clinical practice. Dr. Wang Yanhui, Professor of Xiamen University, created CHB-II-F aiming at cancer patients after chemotherapy and focusing on the nature of the mechanism of the disease caused by chemotherapy drugs. After long-term administration, CHB-II-F can significantly improve the patients’ loss of appetite and the quality of life of the patients (Wang and Xi, 2020). The aim of this study was to study the therapeutic effect and mechanism of CHB-II-F on chemotherapy-induced anorexia in mice with H_22_ hepatocellular carcinoma and to provide evidence for clinical treatment (Figure 9).

In the application of chemotherapy in patients with tumors, chemical drugs can also directly stimulate the gastrointestinal tract at the same time, inhibit and damage the rapid proliferation of gastrointestinal mucosal cells, cause inflammatory changes in the gastrointestinal tract, and reduce the secretion and activity of digestive enzymes. Thus, the digestive and absorption functions of gastrointestinal tract were decreased. The results showed that CHB-II-F combined with 5-FU could enhance the inhibitory effect of 5-FU on the volume and weight of tumor and increase the tumor inhibition rate of 5-FU. CHB-II-F combined with 5-FU can attenuate the pathological damage of gastrointestinal tract induced by 5-FU. The results also showed that CHB-II-F significantly alleviated the loss of appetite induced by 5-FU and increased the weight of tumor-bearing mice after chemotherapy.

In the course of chemotherapy, the balance between free radical production and antioxidant system determines the degree of gastrointestinal injury to a great extent (Hu et al., 2014). MDA is the final metabolite of the process of lipid peroxidation, which is parallel to the degree of lipid peroxidation *in vivo*. Therefore, the content of MDA usually reflects the intensity and speed of lipid peroxidation of free radicals in the body (El-Maghrabey et al., 2014). As the most important free radical scavenger *in vivo*, SOD can protect the tissues from free radical attack. In addition, SOD can catalyze the disproportion reaction of superoxide anion free radicals *in vivo* and can block the toxicity of free radicals and protect the tissue and cellular DNA from oxidative damage (Akbas et al., 2014). Therefore, the activity of SOD and the content of MDA in jejunum can reflect the oxidation and antioxidation of jejunum. The results showed that CHB-II-F could significantly increase the activity of SOD and decrease the content of MDA in jejunum of mice after chemotherapy. Due to increases in free radicals and oxidative metabolites, the damage of gastric mucosa will be aggravated (Zhang, 2012). These results suggest that CHB-II-F may protect the gastrointestinal tract by inhibiting lipid peroxidation, enhancing the activity of gastrointestinal antioxidant enzymes, and maintaining the balance between the production of free radicals and the antioxidant system in the gut, therefore, indirectly improving appetite.

EGF is an endogenous compound, which can increase the synthesis of DNA, RNA, and protein; promote the proliferation of epithelial cells; provide nutrition for gastrointestinal mucosa,; maintain the integrity of gastrointestinal mucosa; and promote the self-healing of gastrointestinal mucosa. In addition, EGF can inhibit gastric acid and promote intestinal peristalsis (Cao et al., 2007). As an important cell growth regulator, PGE_2_ can inhibit gastric acid secretion and the release of cytotoxic substances, increase mucosal blood flow, and play an important role in the repair of gastrointestinal injury. It is an important mucosal protective factor (Kobayashi et al., 2010). GAS and MTL are important excitatory gastrointestinal hormones. GAS not only stimulates gastric acid secretion, but also stimulates the growth of gastric mucosal epithelial cells, which plays an important role in maintaining the integrity of gastric mucosa (Eren et al., 2015). MTL can stimulate both stomach and intestine. In this study, we found that CHB-II-F could increase the levels of EGF, PGE_2_, GAS, and MTL in the serum of tumor-bearing mice after chemotherapy. This suggests that CHB-II-F can attenuate the loss of appetite caused by chemotherapy by stimulating gastrointestinal hormone secretion, accelerating gastrointestinal movement, protecting gastrointestinal mucosal microvessels, increasing mucosal blood flow, and promoting mucosal repair, thus improving gastrointestinal pathological conditions and alleviating gastrointestinal toxicity and side effects caused by chemotherapy.

The hypothalamus is an important center of feeding and energy balance regulation in the body (Abdalla, 2017; Berthoud et al., 2017). The arcuate nucleus (ARC), perifornix area (PFA), lateral hypothalamic area (LH), paraventricular nucleus (PVN), ventromedial hypothalamic nucleus (VMN), and dorsomedial hypothalamic nucleus (DMN) in hypothalamus play an important role in feeding regulation (Varela and Horvath, 2012). The ARC located at the base of the hypothalamus consists of a large number of appetite-promoting neurons (NPY/AgRP) and appetite-suppressing neurons (POMC/CART), which project to other nuclei of the hypothalamus. After integration and processing, the signals pass to the nucleus tractus solitaries (NTS) at the lower center of the brainstem to finally decide whether to continue or stop eating (Lenard and Berthoud, 2008; Morton et al., 2016). Appetite peptides secreted from peripheral organs such as Leptin and Ghrelin can reach the hypothalamic nucleus through the blood-brain barrier and eventually regulate feeding by affecting NPY/AgRP and POMC/CART neurons (Wang et al., 2012).

Leptin is a kind of protein hormone, which is mainly secreted by white adipose tissue, and hypothalamus is the main target of Leptin (Myers et al., 2010). Leptin receptor (Ob-R) is a high affinity receptor of Leptin; Ob-Rb, as the main functional receptor, is widely distributed. It is not only highly expressed in NPY neurons of ARC, but also distributed in orexin neurons and neurons coexpressed by NPY and POMC (Varela and Horvath, 2012; Lee et al., 2018). Leptin is secreted in adipocytes and enters the blood circulation. After passing through the blood-brain barrier, Leptin binds to Ob-R located in the hypothalamus’s ARC to play a role in suppressing appetite and increasing energy expenditure through regulation of the central nervous pathways of POMC/CART and NPY/AgRP (Hillebrand et al., 2002; Sahu, 2003).

Ghrelin is the only hormone in peripheral tissue that promotes appetite and plays an important role in regulating energy balance (Verhulst et al., 2008). It is mainly secreted by X/A-like cells of the gastric fundus acid-secreting gland, followed by blood circulation through the blood-brain barrier, and specifically binds to GHSR, a hunger receptor in the arcuate nucleus of the hypothalamus (Wren et al., 2001). GHSR, mainly located on NPY/AgRP neurons, stimulates eating by stimulating the activity of NPY and AgRP and inhibiting the activity of POMC. Therefore, Ghrelin is considered to be a signal of premeal starvation and initiation of eating (Verhulst et al., 2008; Lv et al., 2018). Some studies have shown that the appetite-promoting effect of Ghrelin can be inhibited by Leptin, and Ghrelin can reverse the suppressive effect of Leptin on appetite. It is suggested that Ghrelin can antagonize Leptin in the appetite-promoting system of NPY/AgRP (Satya et al., 2005).

Orexin secreted from the lateral hypothalamic area is a neuropeptide, which has a strong stimulating effect on food intake. There are two subtypes of Orexin A (OXA) and Orexin B (OXB), both of which are derived from pre-orexin. OX1R and OX2R are two endogenous G-protein coupling receptors of orexin, of which OX1R is the selective receptor of OXA (Sakurai et al., 1998; Garcia-Tornadu et al., 2009). Studies have shown that the nerve fibers emitted by NPY/AgRP neurons can project to orexin neurons, and orexin neurons can also express NPY receptors (Ligang et al., 2011). Therefore, the role of orexin in promoting appetite may be mediated by the NPY/AgRP neural pathway.

Orexin A, Leptin, and Ghrelin bind to their receptors OX1R, Ob-R, and GHSR, respectively, to inhibit or promote appetite by affecting the central NPY/AgRP and POMC/CART neural pathways. The purpose of this study was to investigate the mechanism of promoting appetite by detecting the contents of Orexin A, Leptin, Ghrelin, and NPY in peripheral serum and the expression of GHSR, NPY, POMC, OX1R, AgRP, CART, Ob-R protein, and gene in hypothalamic and by exploring the mechanism of CHB-II-F in promoting appetite. The results showed that CHB-II-F could significantly increase the content of Orexin A, Ghrelin, and NPY in serum and the expression of GHSR, NPY, OX1R, and AgRP protein and gene in hypothalamus and decrease the content of Leptin in serum and the expression of Ob-R, POMC, and CART protein and gene in hypothalamus. These results suggest that CHB-II-F may stimulate NPY/AgRP neurons and inhibit POMC/CART neurons directly or indirectly to attenuate chemotherapy induced loss of appetite by regulating the secretion of Orexin A, Leptin, Ghrelin, and NPY and the expression of GHSR, NPY, POMC, OX1R, AgRP, CART, and Ob-R proteins and genes in hypothalamus.

Nausea and vomiting, as one of the most common side effects of chemotherapy in the gastrointestinal tract, are often an important cause of anorexia (Tina Shih et al., 2007; Grassi et al., 2015; Salihah et al., 2016). The occurrence of chemotherapy-induced nausea and vomiting is a very complex process, and the current study showed that the chemotherapy drugs can stimulate the secretion of 5-HT and SP of the gastrointestinal chromaffin cell. The two neurotransmitters bind the 5-HT_3_ receptor and the NK_1_ receptor, respectively, to generate nerve impulses, which are then transmitted from the abdominal vagus nerve to the chemical trigger zone (CTZ) of the area postrema (AP) located at the bottom of the fourth ventricle, and further to the emetic center, resulting in a vomiting reaction (PJ, 2001; Tsuchiya et al., 2002; Rojas and Slusher, 2012; Rojas et al., 2014). In addition, 5-HT and substance P are also important inflammatory mediators. They can induce the production of TNF- α, IL-6, and other inflammatory cytokines by activating the JNK, a MAPK signaling pathway, thus causing inflammation and aggravating the degree of gastrointestinal mucosal injury (Satheeshkumar and Mohan, 2014). In this study, CHB-II-F could significantly decrease the protein expression of 5-HT and substance P in duodenum. These results suggest that CHB-II-F can reduce gastrointestinal injury caused by 5-HT, inhibit CINV, and relieve chemotherapy induced anorexia.

Due to the complexity of Chinese herbal medicine ingredients and formula combinations, some limitations to our experiments exist. First, as indicated above, it remains to be determined which ingredient(s) in CHB-II-F was responsible for the effects on relieving chemotherapy-induced anorexia. Second, whether CHB-II-F is effective in other HCC lines and models remains to be determined. Finally, although encouraging, the beneficial effects of CHB-II in this animal model of HCC may not be consistent with that in humans.

## Conclusion

CHB-II-F can not only enhance antitumor activity of 5-FU, but also significantly attenuate chemotherapy-induced gastrointestinal side effects, alleviate gastrointestinal injury, regulate gastrointestinal function, promote weight gain, and relieve CINV. Therefore, the mechanism of CHB-II-F in treatment of chemotherapy-induced loss of appetite may be related to its improvement of gastrointestinal injury, regulation of gastrointestinal function, and modulation of both peripheral appetite factor and hypothalamus feeding area of central nervous system.

## Data Availability

The original contributions presented in the study are included in the article/Supplementary Material; further inquiries can be directed to the corresponding authors.

## Abbreviations

AgRP, Agouti gene-related protein; CART, Cocaine and amphetamine regulated transcript peptide; CHB-II-F, Ciji-Hua’ai-Baosheng II Formula; CINV, Chemotherapy-induced nausea and vomiting; CTX, Cyclophosphamide; EGF, Epidermal cell growth factor; ELISA, Enzyme-linked immunosorbent assay; 5-FU, 5-fluorine pyrimidine; GAS, Gastrin; GHSR, Ghrelin receptor; HCC, hepatocellular carcinoma cells; H&E, hematoxylin and eosin; 5-HT, 5-Hydroxytryptamine; MDA, Malondialdehyde; MTL, Motilin; NPY, Neuropeptide Y; Ob-R, Leptin receptor; OXA, Orexin A; OX1R, Orexin receptor 1; PGE2, Prostaglandin E2; POMC, Proopiomelanocortin; RTFQ-PCR, Real-time fluorescence quantitative polymerase chain reaction; SOD, Superoxide dismutase; SP, Substance P; TCM, traditional Chinese medicine; UHPLC, ultra-high performance liquid chromatography; YZXJC, Yangzheng Xiaoji capsule.
