# Supplementary material for: The Ciji-Hua’ai-Baosheng II Formula Attenuates Chemotherapy-Induced Anorexia in Mice With H22 Hepatocellular Carcinoma
Source: Front Pharmacol. 2021 Aug 19;12:715824. doi: 10.3389/fphar.2021.715824 (PMC8416666; doi:10.3389/fphar.2021.715824)
Supplement: Supplementary file 1 [file Table1.docx]

**SUPPLEMENTARY TABLE∣**The identified integrdients in CHB-II-F by UHPLC-HR-ESI-MS

| Structure/Name (no.) | FW | MW | ESI ion peaks in Herb | | | ESI ion peaks in Formula | | |
| --- | --- | --- | --- | --- | --- | --- | --- | --- |
|  |  |  | tR (min) | ESI^+^ * | ESI^-^ | tR (min) | ESI^+^ * | ESI^-^ |
|   naringin | C_27_H_32_O_14_ | 580 | 21.20 | 581.1851 | 579.1715 | 22.21 | 581.1851 | 579.1715 |
|   hesperidin | C_28_H_34_O_15_ | 610 | 22.81 | 611.1945 | 609.1828 | 23.70 | 611.1946 | 609.1829 |
|   5,7,8,4'-tetramethoxyflavone | C_19_H_18_O_6_ | 342 | 35.28 | 343.1171 |  | 35.32 | 343.1171 |  |
|   nobiletin | C_21_H_22_O_8_ | 402 | 35.88 | 403.1383 |  | 35.90 | 403.1383 |  |
|   3,5,6,7,8,3',4'- heptamethoxyflavone | C_22_H_24_O_9_ | 432 | 36.25 | 433.1483 |  | 36.25 | 433.1474 |  |
|   tangeretin | C_20_H_20_O_7_ | 372 | 36.50 | 373.1269 |  | 36.50 | 373.1266 |  |
|   phenylalanine | C_9_H_11_NO_2_ | 165 | 6.61 | 166.0861 |  | 6.71 | 166.0860 |  |
|   salvianic aid A | C_9_H_10_O_5_ | 198 | 8.32 |  | 197.0444 | 8.31 |  | 197.0444 |
|   protocatechualdehyde | C_7_H_6_O_3_ | 138 | 12.63 | 139.0385 | 137.0233 | 12.57 | 139.0388 | 137.0233 |
|   caffeic acid | C_9_H_8_O_4_ | 180 | 15.20 | 181.0491 | 179.0341 | 15.17 | 181.0490 | 179.0341 |
|   lithospermic acid | C_27_H_22_O_12_ | 538 | 23.10 | 539.1176 | 537.1027 | 23.07 | 539.1172 | 537.1050 |
|   rosmarinic acid | C_18_H_16_O_8_ | 360 | 25.21 | 361.0900 | 359.0778 | 25.20 | 361.0915 | 359.0775 |
|   salvianolic acid B | C_36_H_30_O_16_ | 718 | 27.40 | 719.1584 | 717.1470 | 27.32 | 719.1580 | 717.1467 |
|   tanshinone IIA | C_19_H_18_O_3_ | 294 | 35.64 | 295.0963 |  | 35.51 | 295.0961 |  |
|   cryptotanshinone | C_19_H_20_O_3_ | 296 | 36.69 | 297.1471 |  | 36.71 | 297.1482 |  |
|   dihydrotanshinone I | C_18_H_14_O_3_ | 278 | 37.38 | 279.1005 |  | 37.39 | 279.1013 |  |
|   tangshenoside I | C_29_H_42_O_18_ | 678 | 16.85 | 701.2422 [M+Na]+ | 677.2306 |  | 701.2238 [M+Na]+ | 677.2307 |
|   tangshenoside II | C_17_H_24_O_9_ | 372 | 17.83 |  | 371.0987 | 17.81 |  | 371.0986 |
|   kukoamine B | C_20_H_28_O_8_ | 396 | 25.33 | 419.1670 [M+Na]+ |  | 25.29 | 419.1670 [M+Na]+ |  |
|   thalictrine | C_20_H_23_NO_2_ | 341 | 13.37 | 342.1685 | 340.1558 | 13.90 | 342.1695 [M]+ | 340.1553 |
|   spinosin | C_28_H_32_O_15_ | 608 | 19.28 | 609.1796 | 607.1667 | 19.25 | 609.1786 | 607.1661 |
|   nicotinic acid | C_6_H_5_NO_2_ | 123 | 3.41 | 124.0393 |  | 3.36 | 124.0391 |  |
|   pingpeimine B | C_27_H_45_NO_6_ | 479 | 10.00 | 480.3305 |  | 10.35 | 480.3325 |  |
|   pingpeimine A | C_27_H_45_NO_5_ | 463 | 10.12 | 464.3364 |  | 10.50 | 464.3360 |  |
|   pingpeimine C | C_27_H_43_NO_6_ | 477 | 11.23 | 478.3146 |  | 11.60 | 478.3142 |  |
|   peimisine | C_27_H_41_NO_3_ | 427 | 19.51 | 428.3145 |  | 19.97 | 428.3160 |  |
|   peimine | C_27_H_45_NO_3_ | 431 | 20.26 | 432.3454 |  | 20.74 | 432.3470 |  |
|   peiminine | C_27_H_43_NO_3_ | 429 | 21.31 | 430.3293 |  | 21.83 | 430.3306 |  |

* [M+H]^+^, if it is not specified.
